# Canine and Feline Tracheobronchial Foreign Bodies: A UK Multi-Centre Study

**DOI:** 10.3390/ani16050726

**Published:** 2026-02-26

**Authors:** Pedro Alves, Rufus Hammerton, David Walker, Maria Perez, Jessica Florey

**Affiliations:** 1 DWR Veterinary Specialists, Part of Linnaeus Veterinary Ltd., Station Farm, London Road, Six Mile Bottom, Cambridgeshire CB8 0UH, UK; 2 Eastcott Referrals, Part of Linnaeus Veterinary Ltd., Edison Park, Hindle Way, Dorcan Way, Swindon SN3 3FR, UK; 3 Davies Veterinary Specialists, Part of Linnaeus Veterinary Ltd., Manor Farm Business Park, Higham Gobion, Hitchin SG5 3HR, UK; 4 Anderson Moores Veterinary Specialists, Part of Linnaeus Veterinary Ltd., The Granary, Bunstead Barns, Poles Lane, Hursley, Winchester SO21 2LL, UK; 5 Animal Health Trust, Lanwades Park, Kentford, Newmarket CB8 7UU, UK; 6 Cave Veterinary Specialists, Part of Linnaeus Veterinary Ltd., George’s Farm Nr Wellington, West Buckland, Wellington TA21 9LE, UK

**Keywords:** tracheobronchial, airway, foreign body, dogs, cats, endoscopy

## Abstract

Inhalation of foreign material is an uncommon condition that occurs more often in dogs than cats. The main aim of this study was to describe signalment, diagnostic investigation, management and outcomes of dogs and cats with tracheobronchial foreign bodies (TBFBs) in four UK referral centres. Ninety-two dogs and 14 cats with a diagnosis of intraluminal TBFBs between January 2012 and July 2019 were included. Labrador retriever was the most commonly represented canine breed (22/92). Cough was the most common presenting complaint, occurring in 89/92 dogs and 9/14 cats. Summer seasonality was recorded in 74/88 dogs, but no feline seasonality was observed. Radiographic suspected TBFB location agreed with definitive location in 15/22 dogs and 2/2 cats. CT-suspected TBFB location and definitive location agreed in 45/46 dogs and 4/5 cats. Most common TBFB location was right caudal lobe bronchus in dogs (35/97) and trachea in cats (6/14). One of 100 canine TBFBs and nine of 14 feline TBFBs were non-vegetal. Single-attempt bronchoscopic retrieval was successful in 88/92 dogs and 13/14 cats. Surgical retrieval was performed in 4/92 dogs. All patients survived to discharge. This study suggests a pattern of canine TBFB seasonality in the UK. Imaging was useful to guide localisation, and CT appeared more accurate at predicting TBFB location than radiography in dogs. Bronchoscopic TBFB removal was commonly successful, with excellent survival rates. Presenting signs, patterns of seasonality, imaging findings, and management outcomes are useful to help clinical reasoning and decision management in the practical setting.

## Simple Summary

If left untreated, inhalation of foreign material can result in potentially life-threatening complications. The main aim of this study was to describe signalment, diagnostic investigation, management and outcomes of dogs and cats with tracheobronchial foreign bodies (TBFBs) to aid reasoning and decision-making in a clinical setting. Dogs, particularly those of hunting breeds, were more commonly affected than cats, and the most common clinical sign in both species was coughing. Dogs were more commonly affected in summer months and often had a previous history of outdoor exercise. Imaging was useful to guide localisation, with the most common TBFB location being the right caudal lobe bronchus in dogs and trachea in cats. Bronchoscopic TBFB removal was commonly successful with excellent survival rates for both species.

## 1. Introduction

In pets, TBFB aspiration occurs more commonly in dogs than in cats [1], with hunting breeds such as Labrador Retriever and English Setter being over-represented in the literature [1,2,3,4]. TBFBs have been shown to affect 4% of dogs and 8% of cats undergoing bronchoscopy [1]. One study found this value to be as high as 27% in dogs [5].

A myriad of types of TBFBs have been reported in the literature, with vegetal being the most common [1,2,3,4,5,6,7,8]. Other examples include teeth [7,9,10,11,12], stones [8,11,13,14], portions of endotracheal tube [15,16], bones [8,11], and food [17]. The most frequent location of TBFB lodgement is the right-sided bronchus in dogs [1,3,4,5], and trachea in cats [8,11].

Clinical signs can vary depending on the location [8] and severity [11] of airway obstruction. Cough is almost invariably present [3,4,5,6,8,11], being often preceded by outdoor exercise in dogs [3,6]. Dyspnoea, halitosis, haemoptysis, and increased respiratory sounds (e.g., stertor, wheezes) may also be part of the clinical picture [3,4,5,6,8,11].

Diagnostic imaging modalities are often used to confirm the existence and location of a TBFB, with radiography and computed tomography (CT) frequently identifying foreign material or changes associated with its presence [1,4,18,19]. However, due to suboptimal sensitivity and specificity, direct visualization of the airways via bronchoscopy is generally recommended [1,4,18]. Bronchoscopy has been shown to be a successful method for identification and retrieval of TBFBs [1,3,4,5,8].

There is limited literature describing a large number of cases outlining presenting signs, the role of imaging in assisting with TBFB localisation, or case management and outcomes. This information is useful to help clinical reasoning and decision-making in the practical setting. The aim of this study is to describe signalment, diagnostic investigation, management and outcomes of dogs and cats with TBFBs in four UK referral centres.

## 2. Materials and Methods

A retrospective review of patient records of four UK referral centres: Dick White Referrals (DWR, Six Mile Bottom), Davies Veterinary Specialists (DVS, Hitchin), Anderson Moores Veterinary Specialists (AMVS, Winchester), and Animal Health Trust (AHT, Newmarket) was undertaken. Medical records of dogs and cats with a confirmed diagnosis of intraluminal TBFBs presented between 1 January 2012 and 31 July 2019 were reviewed.

Patients were included if a conclusive diagnosis of intraluminal TBFB was made (through direct visualisation via bronchoscopy, or by identification of foreign material within the lumen of the airways in surgically excised tissues) and they had undergone bronchoscopy. Patients were excluded if there were incomplete records of imaging, bronchoscopy procedure, surgical procedure, or referral reports outlining the findings.

The following were recorded: breed, age, sex, clinical signs (including history of outdoor exercise or stick chewing), month of onset, time between onset of clinical signs and presentation to referral practice, physical examination findings, diagnostic tests including biochemistry and haematology where performed, imaging performed at referral centre and respective findings (including suspected location of TBFB based on imaging), method of retrieval, location, and nature and number of TBFBs. Further data analysed included bronchoscopy findings, if broncho-alveolar lavage (BAL) was performed and results of cytology and culture, whether retrieval via bronchoscopy had been attempted at the referring practice, if antibiotics had been given before referral since the start of clinical signs, medication dispensed at referral centre, and survival to discharge.

For the purpose of descriptive analysis, high temperature, fever, and pyrexia were analysed under “pyrexia”; “cough” as its absolute presence, regardless of whether further characterization was described; and hyporexia and anorexia were analysed under “anorexia”. “Increased respiratory sounds” was analysed without discriminating whether it was identified before or after chest auscultation. With regards to bronchoscopy findings: congestion, granulation tissue, hyperaemia, oedema, or erythema were analysed under “inflammation”; pus or purulent discharge analysed under “infection”; mucosal damage or erosion analysed under “ulceration”; and thickened airway as “mural thickening”. Cases of TBFB inhalation in the same patient were considered independent if separated by more than 12 months and complete clinical resolution. Where the number of TBFBs was not specified in the notes, the number of bronchi in which fragments were found was used as the number of TBFBs. Haematology and biochemistry analysis did not discriminate between different panels performed at different institutions. Blood test results, as well as cytology findings, were analysed for the absolute presence of a particular finding.

Diagnostic plan and treatment for an individual case were decided by the resident clinician (under supervision of a board-certified clinician) or the board-certified clinician in charge of that patient. This resulted in several combinations of blood tests, imaging modalities performed, bronchoscopy and BAL methods, and medication used. All imaging findings analysis was based on reports completed by board-certified diagnostic imaging specialists. All cytology findings analysis was based on reports filled by board-certified pathologists. Bronchoscopy was performed by a resident clinician (under supervision of a board-certified clinician) or a board-certified clinician. Bronchoscopic procedures were performed with patients maintained under general anaesthesia by standard protocols. Surgery was performed in those cases where bronchoscopy was deemed unsuccessful by the board-certified clinician in charge of the case.

This was a descriptive study with data manipulation and statistical analysis performed using Excel 2013 (Microsoft Office, Redmond, WA, USA) and Minitab version 21 (Minitab LLC, State College, PA, USA).

## 3. Results

### 3.1. Study Population

A total of 92 dogs and 14 cats matched the inclusion criteria. One dog provided two cases. Cases involved 65 male dogs, of which 38 were neutered, and 27 female dogs, with 14 being neutered. All cats were neutered; seven were male, and seven female. Median (range) age for dog cases was 2.3 years (3 months–11 years), and for cats, 7 years (2–15 years). Canine breeds represented were Labrador Retriever (*n* = 22), crossbreed (*n* = 18, of which four were Labrador crossbreed), Cocker Spaniel (*n* = 13), Springer Spaniel (*n* = 13), German Pointer (*n* = 7), Hungarian Vizsla (*n* = 3), Staffordshire Bull Terrier (*n* = 3), Boxer (*n* = 2), Jack Russell Terrier (*n* = 2), and one each of Australian Shepherd, Bassett Hound, Bichon Frise, Border Collie, Dobermann, English Bull Terrier, French Spaniel, Golden Retriever, and German Shepherd. Thirteen cats were domestic short-haired (DSH), and one was a Siamese crossbreed.

### 3.2. Time of Onset and Duration of Clinical Signs

**Figure 1:**
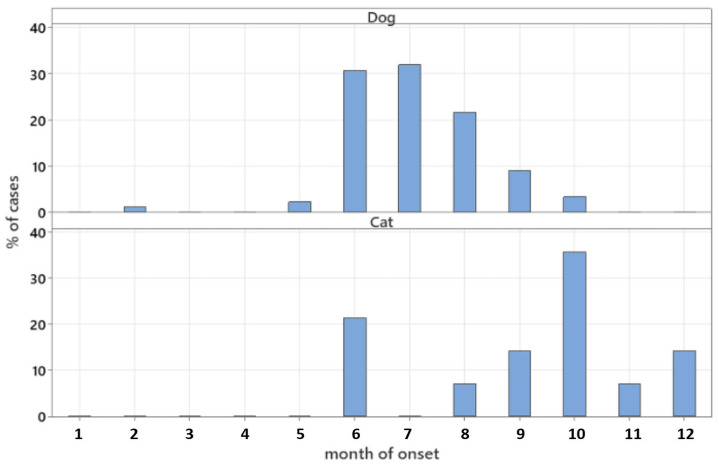
Percentage of dog (**upper**, *n* = 88) and cat (**lower**, *n* = 14) cases by month of onset.

Data on the month of onset of clinical signs were available for 88 dogs and 14 cats and are summarised in Figure 1. Median (range) time interval between start of clinical signs and presentation to referral centre was 13 days (0–240 days) for 85 dogs and 30 days (0–120 days) for all cats.

### 3.3. Clinical Signs and Physical Examination Findings

Reported clinical signs and physical examination findings are summarised in Table 1. For 17/20 dogs for which cough was reported on physical exam, it was specifically elicited on transdermal cervical tracheal pinch. In 45/92 (49%) dogs, there was history of a walk or exercising outdoors preceding the acute onset of clinical signs, mostly 41/92 (45%) in a field, crops, or in the woods. In 5/92 (5%) dogs, there was history of stick chewing preceding the onset of clinical signs. Exercise in a field together with stick chewing was reported in 2/92 (2%) dogs.

### 3.4. Diagnostics

#### 3.4.1. Blood Testing

Blood sampling was performed at the referral centre in 43/92 (47%) dogs. Haematology was performed in 37/43 (86%) cases, packed cell volume (PCV) in 3/43 (7%) cases, serum biochemistry in 42/43 (98%) cases, point-of-care *Angiostrongylus vasorum* antigen detection test in 8/43 (19%) cases, coagulation times in 2/43 (5%) cases, and blood gas analysis in 2/43 (5%) cases. Serum biochemistry was performed in 42/43 cases. Blood tests were performed at the referral centre in 8/14 (57.1%) cats. Haematology and serum biochemistry were performed in 8/8 (100%), point-of-care feline leukaemia virus (FeLV) antigen and antibody to feline immunodeficiency virus (FIV) test in 1/8 (12.5%), and coagulation times in 1/8 (12.5%). In the majority of cases, haematology and biochemistry results were relatively unremarkable.

#### 3.4.2. Radiography and Computed Tomography Findings

Radiographs were performed in 27/92 (29%) dogs. In these 27 dogs, a total of 30 TBFBs were identified in the airways. A specific location was suspected on radiographs for 22/30 (73%) TBFBs. There was agreement between suspected location on radiographs and final TBFB location in 15/22 (68%) TBFBs. In 6/7 (86%) of the cases where no agreement was found, the side (left or right) of the airways where the TBFB was located was the same as suspected on radiographs, and mismatch was due to disparity in specific bronchus location. Radiographs were performed in 3/14 (21%) cats. In these three cats, a total of three TBFBs were identified in the airways. A specific location was suspected on radiographs for 2/3 (66%) TBFBs. There was agreement between suspected location on radiographs and final TBFB location in 2/2 (100%) TBFBs.

**Figure 2:**
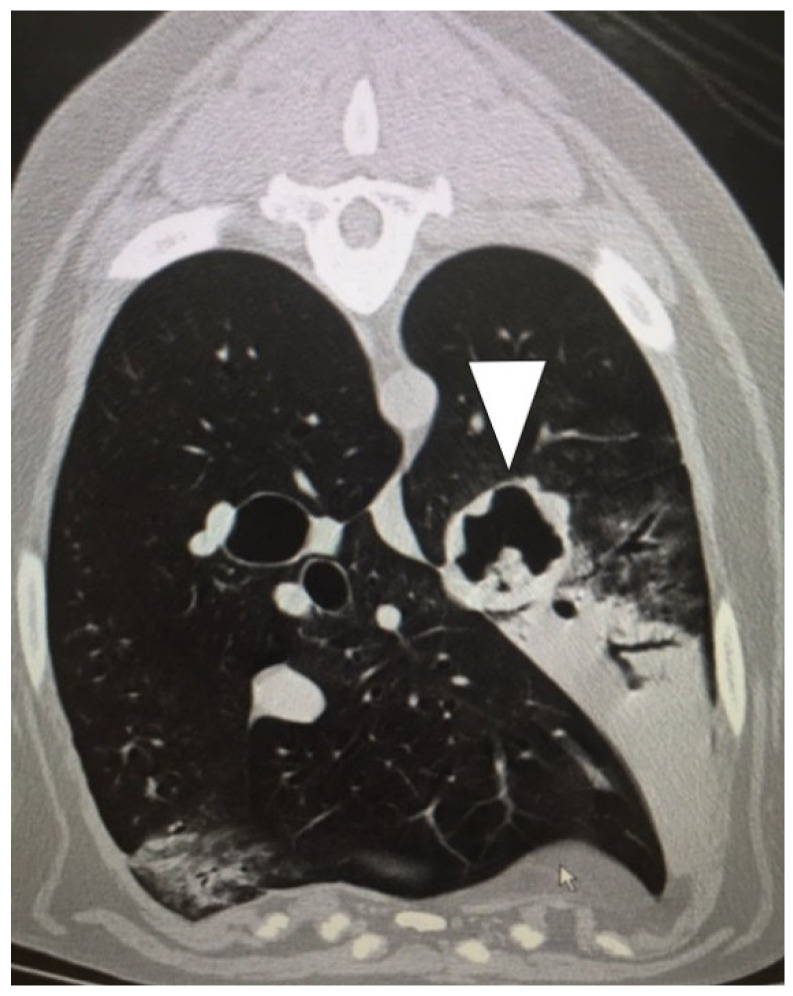
Thoracic CT of a dog, transverse view. Note the presence of bronchiectasis in the left main bronchus and a luminal foreign body (arrow head).

CT was performed in 48/92 (52%) dogs. In these 48 dogs, a total of 50 TBFBs were identified in the airways. Two of these TBFBs were found in the same patient and same location, so they were counted as one for the purpose of imaging accuracy. A specific location was noted on CT for 46/49 (94%) TBFBs (Figure 2). There was agreement between suspected location on CT and final TBFB location in 45/46 (98%) TBFBs. In the case where no agreement was found, the side of the airways wherein the TBFB was located was the same as suspected on CT, and mismatch was due to disparity in bronchus location. CT was performed in 5/14 (36%) cats. In these five cats, a total of five TBFBs were identified in the airways. A specific location was suspected on CT for 5/5 (100%) TBFBs. There was agreement between suspected location on CT and final TBFB location in 4/5 (80%) TBFBs. In the case where no agreement was found, the TBFB was located more cranially (within trachea) than suspected on CT (right main stem bronchus).

Radiographs and CT were both performed in 5/92 (5%) dogs. No information was found stating the criteria used to elect to perform these two types of imaging modalities. No cats had both radiographs and CT.

### 3.5. Bronchoscopy Success and Findings

**Figure 3:**
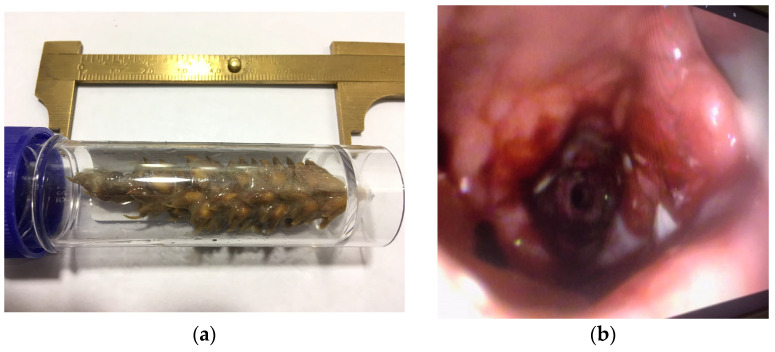
(**a**) Vegetal foreign body (barley stem) retrieved from the patient in Figure 2; (**b**) Bronchial changes seen after retrieval of the foreign body depicted in (**a**). Note the bronchiectasis, inflammation, and mucosal hyperplasia and ulceration.

Bronchoscopy was successful at retrieving airway TBFBs in 88/92 (96%) dogs (Figure 3), with only 1/88 (1%) needing a second attempt to achieve successful TBFB extraction. In 2/88 (2%) dogs, part of a vegetal TBFB was left in the airways. In 4/92 (4%) dogs, TBFB retrieval was successfully achieved surgically. Bronchoscopy was successful at retrieving airway TBFBs in 13/14 (93%) cats. In one (7%) cat, the TBFB (a tooth) was obstructed by a membrane which prevented bronchoscopic retrieval. A surgical approach was recommended but declined by the owner, and the patient was described as treated “conservatively”, although details of what this entailed and on further follow-up were not available. Abnormalities were found on bronchoscopy in 70/92 (76%) of dogs and 12/14 (86%) cats, and are summarised in Table 2.

### 3.6. Foreign Body Description and Location

A total of 100 TBFBs were identified in the airways of 92 dogs. A single TBFB was identified in 85/92 (92%) dogs, with 6/92 (7%) having two TBFBs, and 1/92 (1%) having three TBFBs. In 2/6 cases with two TBFBs, they were located in different bronchi of the same hemithorax, and in 2/6 in bronchi of different hemithoraces. In the case with three TBFBs, all were within the same bronchus (right middle). All 14 cats (100%) had a single airway TBFB.

**Figure 4:**
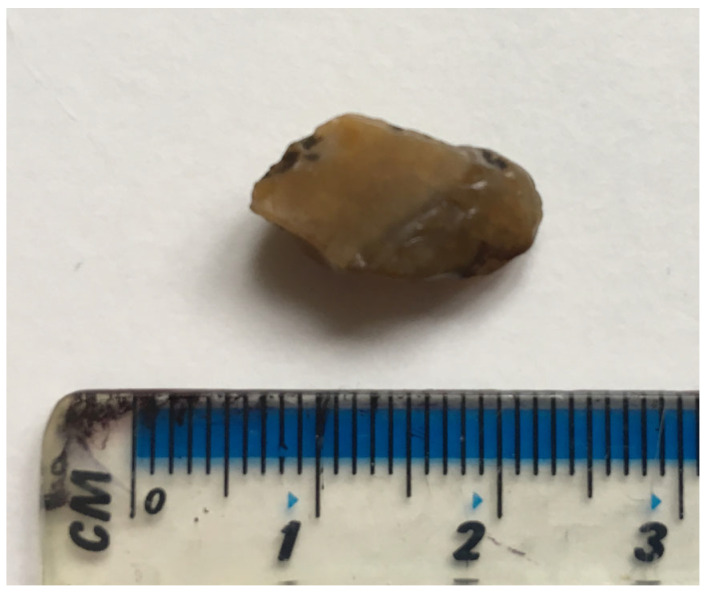
Mineral foreign body (stone) removed endoscopically from the trachea of a cat.

Specific TBFB location was available for 97/100 (97%) TBFBs in dogs and all cat TBFBs (Table 3). In the other 3/100 (3%) canine TBFBs, location was described as “left side” in two and “left secondary bronchus” in one. In dogs, 1/100 (1%) TBFBs was non-vegetal, of unknown nature, and lodged in the right caudal bronchus. In cats, 3/14 (21%) had a mineral TBFB (Figure 4), and 2/14 (14%) had other types of TBFB, specifically a tooth in one case and a fragment of rubber in another. The remainder 9/14 (64%) had vegetal TBFBs.

### 3.7. Bronchoalveolar Lavage Findings

BAL was performed in 16/92 (17%) dogs and in 4/14 (29%) cats. This was always done during bronchoscopy. Cytology was performed in 10/16 (62%) canine samples, and results were available for 9/10 cases. Cytology was performed in 3/4 (75%) feline samples and results were available for 2/3 cases. The findings are summarised in Table 4.

Culture and sensitivity testing were performed in 16/16 (100%) canine samples, and results were available for 14/16 cases. Culture and sensitivity testing were performed in 3/4 (75%) feline samples, and results were available for 2/3 cases. The findings are summarised in Table 5.

### 3.8. Medication Used

#### 3.8.1. Antibiotic Therapy

History regarding antibiotic usage prior to referral was available for 77/92 (84%) dogs and 11/14 (79%) cats. Antibiotics were used at some point between the onset of clinical signs and presentation to referral centre in 44/77 (57%) canine cases and in 9/11 (82%) feline cases. One type of antibiotic was used in 32/44 (73%) dogs, two in 8/44 (18%), three in 3/44 (7%), and five in 1/44 (2%). One type of antibiotic was used in 7/9 (78%) cats, and two in 2/9 (22%). A summary on antibiotic usage can be found in Table 6.

After TBFB retrieval at the referral centre, antibiotics were used as part of treatment in 81/92 (88%) dogs and in 12/14 (86%) cats. Information on which drug used was available in 80/81 canine cases and in all feline cases, and is summarised in Table 7.

There were 77/92 (84%) dogs and 11/14 (78%) cats for which both pre referral and post TBFB removal antibiotic usage history was available. In 8/77 (10%) dogs and 1/11 (9%) cats, no antibiotics were used at any stage of therapy.

#### 3.8.2. Other Medication

Non-steroidal anti-inflammatory drugs (NSAIDs) were used after TBFB retrieval at the referral centre in 50/92 (54%) dogs. NSAIDs used included meloxicam in 35/50 (70%) cases, carprofen in 13/50 (26%), firocoxib in 1/50 (2%), and robenacoxib in 1/50 (2%). Only 1/14 (7%) cats was treated with meloxicam. Glucocorticoid drugs were used after TBFB retrieval in 8/92 (9%) dogs. Drugs used included prednisolone in 6/8 (75%) cases, dexamethasone in 1/8 (12%) cases, and dexamethasone followed by prednisolone in 1/8 (12%) cases. A total of 3/14 (21%) cats were treated with glucocorticoid drugs after TBFB retrieval. Drugs used included prednisolone in 2/3 (66%) cases, and dexamethasone in 1/3 (33%) cases.

### 3.9. Bronchoscopic Retrieval Prior to Referral

Bronchoscopy was attempted at the referring centre in 14/92 (15%) dogs and in 2/14 (14%) cats. In 15 of these patients (all but one cat), a TBFB was identified, but the referring clinician was unable to retrieve it. In an additional feline case, bronchoscopic investigation at the referring practice failed to identify a TBFB which was retrieved by bronchoscopy at the referral centre.

### 3.10. Outcomes

All dogs and cats included in this study survived hospitalisation and were deemed healthy to be discharged.

## 4. Discussion

In our study, bronchoscopy was highly successful at retrieving airway TBFBs in dogs and cats, with only 4% and 7% of cases warranting surgical treatment, respectively. Impaired visualisation of the TBFB by blood or mucus or TBFB localisation beyond the reach of the bronchoscope have been reported as reasons for failed endoscopic retrieval [1,3,4]. Indeed, in one of the cases that required surgical therapy, the narrow lumen of the airways and the presence of mucus had prevented bronchoscopic TBFB visualisation, and in two of the others, unsuccessful retrieval had been due to the inability of the bronchoscope to reach the area where the TBFB was lodged. Nevertheless, the success rate observed is comparable to that of other canine [1,3,4,20], feline [8], and human [21,22] studies, and all of the patients survived to discharge. However, it is important to highlight that our findings are limited by the fact that length of hospitalisation and short- and long-term follow-up (e.g., regarding development of complications and/or persistent clinical signs) were not investigated. Additionally, this study excluded all cases where foreign bodies were not identified in the lumen of the airways (such as migrating foreign bodies, which may have had different outcomes), thus limiting its generalisability to all airway foreign body cases. Despite the high retrieval success rate observed in this study, in 15% of dogs and 7% of cats, the referring primary care clinician was unable to retrieve the TBFBs after achieving bronchoscopic identification. The retrospective nature of this study precluded clarification on the reasoning behind this finding, but it may have been due to operator expertise, equipment quality, TBFB movement/dislodgement, or a combination of these. In a recent study, 11% of dogs with vegetal TBFBs had a previous bronchoscopic examination by a referring veterinarian, and in 55% of those cases, the TBFB was visualised but removal was unsuccessful [4]. Although endoscopic retrieval has been shown to have a high success rate, the possibility of retrieval failure should therefore still be discussed with clients prior to attempting retrieval.

In this study, we found a predominance of canine TBFB cases, with a total of 92 canine cases versus 14 feline cases. The case distribution seen may have been influenced to an extent by the overall canine and feline population seen at the hospitals from which data were collected. Nevertheless, the species distribution observed is similar to that found in the literature, wherein greater numbers of canine TBFB cases are reported when compared with feline [1,3,4,5,8,11]. Furthermore, a study found TBFBs to account for 8% of all cases of dogs undergoing a bronchoscopic procedure, twice that observed in cats (4%) [1]. We found TBFBs affected dogs and cats from a wide range of ages (3 months to 11 years in dogs and 2 to 15 years in cats), as previously reported [1,3,4,8,11]. We also observed a greater number of male dogs with TBFBs, as found in the literature [1,3]. No sex predominance was observed in cats, consistent with previous studies [8,11].

Studies have found hunting dog breeds to be over-represented in cases of TBFBs, specifically Labrador retrievers [1,2] and English setters [3,4]. Our findings are consistent with those found in the literature, with three of the four most commonly represented breeds being hunting dog breeds, specifically Labrador retriever, cocker spaniel, and springer spaniel. However, these breeds’ representation may be influenced by their popularity and might reflect the general population in the referral centres of our study. Therefore, further studies would be needed to assess breed-specific risk by comparing cases with a control population, ascertain what proportion of these dogs are actually used as hunting dogs, and whether TBFB aspiration could have occurred during hunting-related activities. In our study, we found that for 49% dogs, there was history of a walk or exercising outdoors preceding the onset of clinical signs, with outdoor activities occurring specifically in a field, crops, or in the woods in 45% of cases. It was not possible to determine if these were related to hunting activities. A study found that all 41 dogs with TBFBs had a history of outdoor activity preceding the onset of clinical signs [3]. Despite the disparity between that study and our findings, we believe this is important clinical history information that should be collected, particularly in a coughing patient. The observed disparity may, in part, result from an underestimation of our values, resulting from the retrospective nature of this study. In cats, DSH represented 93% of the cases in our study, which is similar to the literature, wherein the majority of cases reported occurring in DSH or domestic long-haired cats [8,11]. This is likely to be due to the higher numbers of DSH in the overall feline population.

Onset of clinical signs occurred during summer months (June, July, and August) in 84% of the dogs of this study. No particular seasonal variation was noted in cats. Seasonal variation has been reported in dogs and cats with migrating intrathoracic grass awns, with 70% of cases presenting during spring or summer months [18]. A different study has also suggested summer seasonal variation to occur in dogs and cats with grass seed foreign bodies [19]. Summer seasonal variation has been observed in a small UK-based canine case series of intrabronchial TBFBs in dogs [6], and in a case series from France, wherein 88% of cases had an onset of clinical signs during spring and summer months [4]. This study presents further evidence supporting seasonal variation in TBFB aspiration in dogs. With vegetal TBFBs representing 99% of those found in dogs in our study, it is inevitable to relate seasonal variation with TBFB type. It appears likely that an environmental increase in potential vegetal TBFBs such as grass awns or seeds during summer months, alongside a potential increase in outdoor activities, would be directly related to the higher number of cases of aspiration during the same time, as suggested elsewhere [4]. Further studies would be needed to corroborate these speculations.

Clinical signs resulting from TBFBs lasted for hours to several months before presentation to referral centre in both dogs and cats, consistent with previous studies [1,4]. In these studies, the median duration of clinical signs by the time of presentation was 21 and 30 days. Similarly, our study demonstrated a significant, though slightly shorter, time period between onset of clinical signs and presentation, with a median of 13 days. In cats, one study found that the majority presented with at least 2 weeks’ history of clinical signs, 33% with signs that lasted 2–4 weeks, and 33% with signs lasting over 4 weeks [8]. These are relatively comparable to our study, as the median duration of clinical signs in cats by the time of presentation was 30 days. As previously reported [3,4,5,6,8,11], cough was the most common clinical sign found in both dogs and cats. Cough elicited with mild tracheal palpation is often reported on physical examination in cases of canine respiratory infectious disease complex (“kennel cough”) [23]. In our study, for 17/20 dogs for which cough was reported on physical exam, it was specifically elicited on cervical tracheal palpation. This emphasises the importance of not overlooking TBFBs as a relevant differential diagnosis when that examination finding is identified. Pyrexia has been reported associated with TBFBs in dogs and cats, but it appears to be uncommon [4,6,8,19]. Our findings are consistent with the literature, with pyrexia being uncommon in both canine and feline cases. In our study, dyspnoea was more commonly found in cats than dogs. Although robust interspecies comparisons are limited by the stark difference in case numbers, the prevalence of feline dyspnoea in our study (29%), as opposed to canine (5%), is more similar to that found in the larger human case series (13–31%) [21,24]. This is interesting considering that TBFBs were lodged in the trachea in 43% of the cats, whereas in humans, tracheal TBFBs only made up 10.5–13.2% of cases [21,24], perhaps suggesting that TBFB location may have a different impact on the development of dyspnoea in these species. Dyspnoea prevalence in the cats of our study was lower than found in other feline case series, wherein it was present in 75% of cats with TBFBs [8], and in 100% of cats with tracheal TBFBs [11]. Ultimately, as previously suggested [11], feline bronchi of a smaller diameter may make tracheal TBFBs relatively more common in cats than in dogs. Mineral TBFBs will frequently lodge in the trachea of cats, leading to dyspnoea [8,11]. In the cats of this study, while all mineral TBFBs were lodged in the trachea and resulted in dyspnoea, they represented 75% of cases of dyspnoea and only 21% of all cases of feline TBFBs. The lower prevalence of feline dyspnoea found may therefore be a result of the morphology of the TBFBs aspirated by the cats of this study.

Thoracic radiography is a well recognised method to identify and localise TBFBs in dogs and cats. Agreements of 33–66% between suspected location on radiographs and final TBFB location have been reported for both dogs and cats [1,20], but this agreement can be as high as 92–100% in exclusively feline case series [8,11]. These findings are similar to those of our study for both dogs (68%) and cats (100%) and show that although thoracic radiography is helpful in making a diagnosis, it does not replace a complete bronchoscopic examination. Despite the potential usefulness of radiography, only 29% of dogs and 21% of cats had thoracic radiographs taken in the referral setting (when compared with 52% and 36% that had CT, respectively). There could be several factors influencing imaging modality selection, including imaging and/or endoscopic diagnosis having been performed prior to referral, CT availability, individual clinician preference, perceived disease severity, or a combination of these. Previous work has shown that CT allows the detection of more abnormalities, the correct site of the abnormality, and the path of a migrating thoracic foreign body more accurately than radiography [18]. Indeed, in our study, a specific location was suspected on CT in 94% and 100% of canine and feline TBFBs, respectively, and a 98 and 80% agreement between suspected location on CT and final TBFB location in dogs and cats, respectively, was identified. This is similar to what has been reported in dogs, wherein CT scan localization of the TBFB was confirmed by endoscopy in 88% of cases [4]. It is, however, important to consider that many vegetal TBFBs may not be visualised on CT and that CT lesions may be seen at sites without a vegetal TBFB [4,19].

Vegetal TBFBs comprised all but one of the canine cases and were most commonly found in the right hemithorax (57%). This differs from what has been reported by Vansteenkiste et al. [19] but is similar to what has been reported in several other studies [1,2,3,4,5,20]. Vegetal were the most common type of TBFB identified in cats, followed by mineral. TBFBs were more commonly found in the bronchial tree (57%) than in the trachea (43%). These observations differ from those of Leal et al. [8] (equal frequency) and Tenwolde et al. [1] (more frequent in the trachea). Tenwolde et al. [1] had hypothesised that their findings might have been explained by the smaller diameter of the feline airways, whereas Leal et al. [8] suggested that the nature and shape of a TBFB may dictate its final location. Interestingly, in the latter study, no statistical relationship was found between TBFB nature and its location. One of the limitations of our study is the small number of feline cases. This may explain the disparity observed between ours and the previous studies, and it may be that with a higher number of cases, more definite conclusions can be drawn. Nevertheless, as seen in dogs, when feline TBFBs were located in the bronchial tree, the right hemithorax was more prevalent. Therefore, it appears evident that, for both species, once inhaled, TBFBs will most likely follow the path of least resistance, which will likely lead to the right main bronchus given its straighter alignment with the trachea when compared with the left, as previously suggested [1,4,8].

For dogs where two or more TBFBs were identified, we found that these were located in different hemithoraces in 29% of cases. This highlights the importance of performing a full bronchoscopic examination of the bronchial tree despite the fact that having multiple TBFBs was a less common occurrence (8–30% of cases), as previously suggested [3,4].

Inflammation, infection, mucus accumulation, and haemorrhage were the most common alterations identified on bronchoscopy in both dogs and cats. These are non-specific findings that can be identified in multiple airway conditions in both dogs and cats [25,26,27], and are likely due to a combination of direct irritation/trauma caused by the TBFB and transport of infectious agents. In a recent work, a large bronchial nodule or irregular mucosa were identified in 82% of cases of canine vegetal TBFBs [4]. This has not been reported as a finding in our study. The reason for this discrepancy remains uncertain, but it may, to an extent, be an underestimation of the changes seen, due to inter- and intra-clinician variation in lesion description, lack of standardisation in lesion description, or a combination of these. This would be supported by the frequent use of broad descriptions such as “inflammation”, as well as terms such as “mucosal hyperplasia” or “ulceration” that may represent potentially similar findings, disregard grading of severity, and, in some cases, would ideally require histopathological confirmation. These factors make meaningful analysis of our bronchoscopic findings notably challenging. Flageollet et al. [4] suggested that the bronchial nodule or irregular mucosa could be directly related to chronic friction between the TBFB and mucosa and chronic mucosal inflammatory reaction. Standardisation and more detailed lesion description in future prospective investigation may prove beneficial to clarify the association between these cases’ chronicity and the bronchoscopic findings. Despite the low number of cases for which cytological BAL results were available, septic neutrophilic inflammation was indeed the most common finding observed, as previously reported [1]. *E. coli* was the most common (43%) microorganism identified on canine BAL cultures, although it accounted for only 22% of cases in a different study [1]. This difference may be due to the small number of cases in our study which had BAL and culture performed.

One of the main limitations of this retrospective study is the fact that the diagnostic plan and treatment for an individual case were decided by the clinician in charge of that patient. This resulted in different combinations of diagnostics, from imaging modalities performed to medication used, including antibiotic choices. In an era where responsible antibiotic prescription is of paramount importance, appropriate case and antibiotic therapy selection are essential. In our study, interpretation of data related to antibiotic use is limited by the lack of standardized protocols, limited BAL cytology and culture data, and the fact that antimicrobial decision-making could not be systematically assessed. Furthermore, it was not clear to what extent BAL cytology and culture results influenced antibiotic selection, or whether they were primarily used therapeutically or prophylactically. Inhaled TBFBs can carry infectious agents which can potentially result in pneumonia [27]. In our study, antibiotics were used at some point between the onset of clinical signs and presentation to the referral centre in 57% of canine cases and 82% of feline cases. In 88% of dogs and 86% of cats, antibiotics were used after TBFB retrieval at the referral centre, with the most common drug selected being potentiated amoxicillin. Bronchoscopy and BAL findings are some of the factors that can influence antibiotic usage [1,3] but this appears to be ultimately clinician-dependent. Only 10% of dogs and 9% of cats in our study had no history of antibiotic usage, and all survived to discharge, although collecting post-discharge follow-up information was beyond the scope of this study. Successful outcomes without antibiotic usage have been reported elsewhere [11], and it may be that acute uncomplicated cases do not require antimicrobial therapy. In children, antibiotic usage was reported in only 55% of cases of airway foreign bodies retrieved with flexible endoscopy [21]. Looking specifically into the antibiotic usage in cases of TBFB inhalation was beyond the scope of our study, but it was interesting to observe how frequent they were part of the therapeutic plan. Perhaps further studies may clarify which cases of TBFB inhalation warrant antibiotic therapy. If required, antimicrobial usage should ultimately be guided by sensitivity results and prudence guidelines [28].

## 5. Conclusions

This study offers a detailed look into the signalment, diagnostic investigation, management, and outcomes of dogs and cats with TBFB in the UK. Dogs, particularly those of hunting breeds, appear to be more commonly affected than cats, and our results suggest a pattern of canine TBFB seasonality in the UK. Imaging was useful to guide localisation but should not replace complete bronchoscopic examination of the bronchial tree. Although long-term follow-up data were not reviewed, bronchoscopic TBFB retrieval was commonly successful, with excellent survival rates at discharge.

## Figures and Tables

**Table 1:** Frequencies (and percentages) of clinical signs and physical examination findings in dogs and cats presenting with tracheobronchial foreign bodies.

	Cat *n* = 14		Dog *n* = 92	
Type of Clinical Sign/Finding	Physical Exam	Clinical Signs	Physical Exam	Clinical Signs
Cough	1 (7%)	9 (64%)	20 (22%)	89 (97%)
Haemoptysis	-	-	-	13 (14%)
Retching	1 (7%)	-	1 (1%)	12 (13%)
Lethargy	-	1 (7%)	2 (2%)	7 (8%)
Anorexia	-	1 (7%)	-	6 (6%)
Gagging	-	-	-	5 (5%)
Pyrexia	1 (7%)	-	6 (6%)	4 (4%)
Tachypnoea	2 (14%)	2 (14%)	7 (8%)	4 (4%)
Regurgitation	-	-	-	2 (2%)
Sneezing	-	1 (7%)	-	2 (2%)
Vomiting	-	-	-	2 (2%)
Weight loss	-	-	-	2 (2%)
Blood in saliva	-	-	-	1 (1%)
Cutaneous eruptions	-	-	-	1 (1%)
Halitosis	-	-	-	1 (1%)
Diarrhoea	-	-	-	1 (1%)
Dyspnoea	4 (29%)	4 (29%)	5 (5%)	-
Increased respiratory sounds	6 (43%)	2 (14%)	11 (12%)	-
Exercise intolerance	-	1 (7%)	-	-
Low body condition score	-	-	4 (4%)	-
Decreased respiratory sounds	1 (7%)	-	2 (2%)	-
Heart murmur	-	-	2 (2%)	-
Hypersalivation	-	-	1 (1%)	-
Increased capillary refill time	-	-	1 (1%)	-
Oedematous muzzle and forelimbs	-	-	1 (1%)	-
Pale mucous membranes	-	-	1 (1%)	-
Tachycardia	-	-	1 (1%)	-

**Table 2:** Frequencies (and percentages) of abnormalities found on bronchoscopy in dogs and cats.

Endoscopy Findings	Cat *n* = 14	Dog *n* = 92
Inflammation	7 (58%)	51 (73%)
Infection	4 (33%)	26 (37%)
Mucus	4 (33%)	11 (16%)
Bronchiectasis	-	10 (14%)
Haemorrhage	2 (17%)	10 (14%)
Mucosal ulceration	1 (8%)	6 (9%)
Bronchial mural thickening	-	2 (3%)
Mucosal hyperplasia	1 (8%)	1 (1%)
Mucosal necrosis	-	1 (1%)
Bronchial collapse	1 (8%)	-
Membrane covering the TBFB	1 (8%)	-

**Table 3:** Frequencies (and percentages) of location of 97 dog and 14 cat foreign bodies.

TBFB Location	Cat TFBF *n* = 14	Dog TFBF *n* = 97
Right caudal bronchus RCd	3 (21%)	35 (36%) ^1^
Right middle RM	1 (7%)	11 (11%)
Right accessory RA	-	8 (8%)
Right main Rmain	1 (7%)	2 (2%)
Left caudal bronchus LCd	1 (7%)	29 (30%)
Left cranial LCr	1 (7%) ^2^	8 (8%)
Left main Lmain	1 (7%)	4 (4%)
Trachea	6 (43%) ^3^	-

^1^ One non-vegetal; ^2^ Tooth; ^3^ Four non-vegetal (three mineral, one rubber).

**Table 4:** Frequencies (and percentages) of cytological bronchoalveolar lavage findings in dogs and cats with TBFBs.

Type of Inflammation	Cat *n* = 2	Dog *n* = 9
Septic neutrophilic inflammation	1 (50%)	6 (67%)
Non-septic neutrophilic inflammation	-	1 (11%)
Non-septic mixed inflammation with eosinophils	-	1 (11%)
Neutrophilic inflammation with eosinophils suspected septic	-	1 (11%)
Non-specific inflammation	1 (50%)	-

**Table 5:** Frequencies (and percentages) of microorganisms identified on bronchoalveolar lavage culture of dogs and cats with TBFBs.

Microorganism Grown	Cat *n* = 2	Dog *n* = 14
*E. coli*	-	6 (43%)
*Pseudomonas aeruginosa*	-	2 (14%)
*Streptococcus* spp.	-	2 (14%)
*Alcaligenes faecalis*	-	1 (7%)
*Candida* spp.	-	1 (7%)
*Enterococcus faecalis*	-	1 (7%)
*Morganella morganii*	-	1 (7%)
Non-specified anaerobes	-	1 (7%)
*Pasteurella multocida* and *Prevotella* spp.	1 (50%)	-
No growth	1 (50%)	4 (29%)

**Table 6:** Number (and percentages) of each type of antibiotics used in dogs and cats with TBFBs prior to referral.

Type of Antibiotic	Cat *n* = 9	Dog *n* = 44
Amoxicillin-clavulanate	3 (33%)	23 (52%)
Doxycycline	1 (11%)	7 (16%)
Amoxicillin	1 (11%)	4 (9%)
Marbofloxacin	1 (11%)	4 (9%)
Cephalexin	-	3 (7%)
Metronidazole	-	3 (7%)
Trimethoprim-sulfa	-	3 (7%)
Cefuroxime	-	1 (2%)
Enrofloxacin	-	1 (2%)
Oxytetracycline	-	1 (2%)
Pradofloxacin	-	1 (2%)
Cefovecin	4 (44%)	-

**Table 7:** Number (and percentages) of antibiotic usage in dogs and cats after TBFB removal in a referral setting.

Type of Antibiotic	Cat *n* = 12	Dog *n* = 80
Amoxicillin-clavulanate	7 (58%)	61 (76%)
Cephalexin	1 (8%)	11 (14%)
Doxycycline	1 (8%)	3 (4%)
Marbofloxacin	2 (17%)	2 (2%)
Amoxicillin-clavulanate and metronidazole	-	2 (2%)
Amoxicillin-clavulanate and marbofloxacin	-	1 (1%)
Pradofloxacin	1 (8%)	-

## Data Availability

The data are contained within the article. The data presented in this study are available in the tables and figures in the article “Canine and feline tracheobronchial foreign bodies: a UK multi-centre study”.
